# Evaluation of Growth Curve Models for Body Weight in American Mink

**DOI:** 10.3390/ani10010022

**Published:** 2019-12-20

**Authors:** Duy Ngoc Do, Younes Miar

**Affiliations:** Department of Animal Science and Aquaculture, Dalhousie University, Truro, NS B2N5E3, Canada; duy.do@dal.ca

**Keywords:** body weight, growth curve, mink, non-linear models

## Abstract

**Simple Summary:**

Understanding the animal growth is important for optimized management and feeding practices as well as genetic improvement of animals; however, little is known about the growth of mink raised in Canada. This study evaluated the performances of ten models to find the best models describing the growth curves in mink. The results showed that Logistic and Richards were the best model for males and females, respectively. Growth curves were different between males and females. These results suggested that Richards model can be used for modelling the mink growth and modelling might be performed separately for male and female individuals.

**Abstract:**

Modelling the growth curves of animals is important for optimizing the management and efficiency of animal production; however, little is known about the growth curves in American mink (*Neovison vison*). The study evaluated the performances of four three-parameter (Logistic, Gompertz, von Bertalanffy, and Brody), four four-parameter (Richards, Weibull, Bridges, and Janoscheck) and two polynomial models for describing the growth curves in mink. Body weights were collected from the third week of life to the week 31 in 738 black mink (373 males and 365 females). Models were fitted using the *nls* and *nlsLM* functions in stats and minpack.lm packages in R software, respectively. The Akaike’s information criterion (AIC) and Bayesian information criterion (BIC) were used for model comparison. Based on these criteria, Logistic and Richards were the best models for males and females, respectively. Four-parameter models had better performance compared to the other models except for Logistic model. The estimated maximum weight and mature growth rate varied among the models and differed between males and females. The results indicated that males and females had different growth curves as males grew faster and reached to the maximum body weight later compared to females. Further studies on genetic parameters and selection response for growth curve parameters are required for development of selection programs based on the shape of growth curves in mink.

## 1. Introduction

Understanding the animal growth is important for optimized management and feeding practices as well as genetic improvement of the species. American mink (*Neovison vison*) is a medium size member of the Mustelidae family [1], which is often used for its fur. Mink are often born in late April or early May and are pelted in late November–December [2,3]. The mature size of mink depends on the annual change in the circadian rhythm of day length. Therefore, obtaining curves that adequately represent their growth will help mink farmers to modify the feeding practices and develop the selection program for better growth performance and fur quality. In fact, mink breeding in Canada is currently based on the phenotypic selection of body size, reproductive performance and fur quality [3]. 

Growth curves are used to describe the changes in body mass or length or number of cells over time. Modelling of growth curves is particularly useful because it provides means for visualizing growth patterns over time, and the generated equations can be used to predict the expected weight of group of animals at specific age [4]. The shape of growth curve can be used in the selective breeding programs [5]. In animal species, growth parameters were shown to be heritable and responsive to the selection programs [6,7,8,9]. A number of growth equations have been used to fit the growth curve of livestock, among them non-linear mathematical models, such as Gompertz, Brody, Von Bertalanffy, Logistic, and Richards are widely used to describe the growth curve [6,7,8,9,10,11]. These non-linear models allow the interpretation and understanding of growth patterns and metabolism underlying growth periods [5]. Few studies have been devoted to model the growth curve in mink [12,13,14,15]. Using the multiphasic growth model, Sørensen et al. [13] indicated that growth curve parameters in mink are significantly associated with gender and feed efficiency level. Liu et al. [12] indicated that mink colors are important for model selection since performance of growth models varied among the mink colors. The authors compared six different models and suggested that Logistic, Gompertz, and Richards models are suitable for assessing the growth pattern of male mink [12]. However, this research is only focused on male mink [12], it is important to assess the model performance in female mink. In fact, Rong et al. [15] observed the better performance of Gompertz models compared to Logistic models when analyzing data obtained from three breeds of MingHua black, JinZhou black, and Pastel. The main objective of this study is to evaluate the performance of growth curve models for male and female black mink in the Canadian populations. 

## 2. Materials and Methods 

### 2.1. Animal Resources, Data Collection, and Editing 

The proposed work was approved by the Dalhousie University Animal Care and Use Committee (certification# 2018-009), and mink used in this study were cared for according to the Code of Practice for the Care and Handling of Farmed Mink (https://www.nfacc.ca/pdfs/codes/mink_code_of_practice.pdf) guidelines. Samples were collected from Millbank Fur Farm (Rockwood, ON, Canada). Mink were housed under standard farming conditions, and diets were adjusted according to animal requirements in each production period. Each annual cycle of mink reproduction was started by mating between males and females on the beginning of March. Females were moved into the male pens for breeding. Mink were randomly selected for breeding in November or early December. This study included 796 mink kits that were born by mating 63 healthy sires and 135 healthy dams. These animals were born in the mid-spring (1 May 2018) and were weaned around 1 July 2018. All mink were black and they were free from Aleutian disease. Two mink (a male and a female) were kept in a cage and were fed *ad libitum* with the commercial feed, which was by-products from human food production. The feed ingredients varied in breeding, growth, and furring periods depending on the availability of feed sources (Table S1). The chemical compositions and metabolic energy of mink diet over different periods were listed in Table 1. Mink were weighed eight times every four weeks from the third week after birth (22 May 2018) to the harvesting day (10 December 2018). Body length was measured at harvesting day (10 December 2018). For quality control of data, mink with less than five records of body weight were excluded. The final data including 738 mink (373 males and 365 females) was used for testing the growth model performance. Prior to fitting growth curve models, we examined the significance of fixed effects including weeks, sex, and their interactions on body weight using the following linear mixed model:

BW_ijk_ = μ + w*_i_* + s*_j_* + w_i_*s*_j_* + a*_k_* + *e_ijk_* (1)

where BW_ijk_ is the vector of body weight at week i of animal k with sex j_,_ μ is the overall mean, w_i_ is the vector of fixed effect of week i, s_j_ is the vector of fixed effect of sex j, w_i_*s_j_ is the interaction between week i and sex j, a is the vector of random effect of animal k, and *e_ijk_* is the random error. The differences between estimated means of body weight for males and females by week were calculated using the lmerTest package in R software (R Core Team, Vienna, Austria) [16] and significant differences were declared at *p* < 0.05. 

### 2.2. Growth Modelling and Evaluations 

A selection of eight different non-linear growth models including four three-parameter models and four four-parameter models were used for modelling of growth curves in males and females separately. The three-parameter models included Logistic [17], Gompertz [18], Von Bertalanffy [19], and Brody [20]. The four-parameter models included Weibull [21], Richards [22], Bridges [23], and Janoscheck [24] (Table 2). Moreover, two linear models with polynomial structure (third degree polynomial and fourth degree polynomial) were used to compare with these aforementioned models. 

In each model, the body weight (BWt) was fitted as a function of week of measurement. All models were fitted using *nls* or *nlsLM* functions in stats and minpack.lm packages in R software (R Core Team, Vienna, Austria), respectively [26]. The Akaike’s information criterion (AIC) and Bayesian information criterion (BIC) were chosen to determine the most optimal model. The AIC and BIC were chosen because likelihood ratio tests tend to favor models with multiple parameters and lower values of AIC and BIC reflect better fitting. The AIC and BIC were defined as

AIC = −2 log-Likelihood + 2K

BIC = −2 log-Likelihood + KN

where log-Likelihood is the maximum likelihood, K is the number of parameters in the model and N is the sample size. 

## 3. Results 

The descriptive statistics for body weights of mink males and females from week 3 to week 31 are shown in Table 3. The maximum body weight of mink was observed at week 31 (3.10 ± 0.36 kg) and week 19 (1.63 ± 0.20) for male and female mink, respectively. The Pearson correlations between body weights at different age and body length at harvest were from moderate (before weaning: week 3 and 7) to high (after weaning). Based on the results from linear mixed model tests, sex, week, and their interactions had significant effects (*p* < 0.05) on body weights (Table S2). The estimated least square means of body weights of males were significantly (*p* < 0.05) higher than the values of females in all weeks, except for week 3 (Table S2).

The goodness of fit of ten models is shown in Table 4. Based on the AIC and BIC criteria, the Brody was the worst models as it had the highest AIC and BIC in both males and females. While Logistic model was the best model based on AIC or BIC in males as it had the lowest AIC and BIC values compared to the other models (AIC = 756.21; BIC = 780.03). The Richards had the lowest AIC and BIC values compared to the other models in females (AIC = −1587.20; BIC = −1557.52). Two polynomial models were worse than four-parameter models. Except for Logistic model, other three-parameter models had worse performance than four-parameter models, regardless the data used. The Weibull, Bridges, and Janoscheck had the same values for both AIC and BIC in both males and females. The negligible differences in AIC and BIC values between these models with the values obtained from Richards model in females suggested that all of four-parameter models are equally performed for females.

The estimated parameters for each model are shown in Table 5. The estimated maximum body weights (α) varied among the models and between males and females. The highest estimated mature weights (α) were observed in Brody models with values of 3.49 kg for males and 1.77 kg for females (Table 5). The lowest estimated mature weights were observed in the Bridges model (2.88 kg for males and 1.53 kg for females). The estimated mature growth rate (k) also varied among the models and ranged from 0.09 ± 0 in Brody models to 2.31 ± 0.04 in Logistic model for males and from 0.10 ± 0.01 in Bridges and Janoscheck models to 2.28 ± 0.04 in Logistic model for females. 

**Figure 1:**
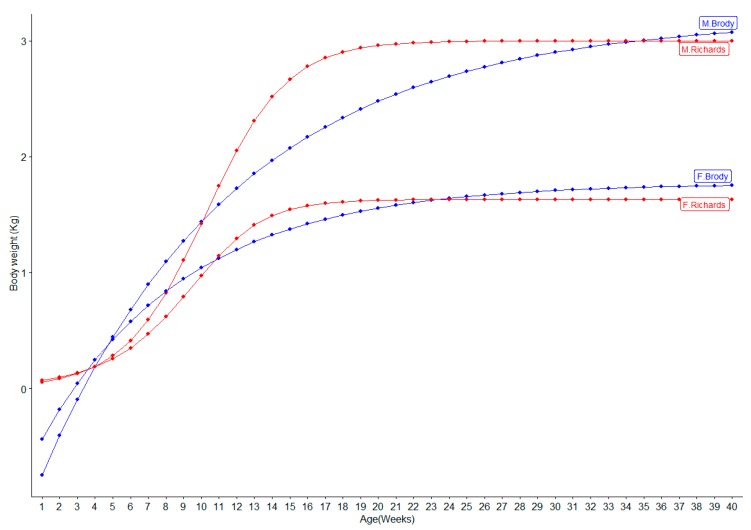
The growth curve of mink based on the best (Richards), and the worst (Brody) model. The blue line showed the predicted values for Brody model in males (M.Brody) and females (F.Brody) and the red line showed the predicted values for Richards model in males (M.Richards) and females (F.Richards).

Predicted growth curves from the best (Richards) and worst models (Brody) are shown in Figure 1. Males were predicted to reach to their mature weight at week 20 while females were predicted to reach their mature weight earlier (at week 16) based on Richards model. The highest increase in the body weight appeared around week 5 to 16 in males and around week 5 to 14 in females in Richards model, which was close to the change observed in the actual data as described above. In t Brody model, body weight was predicted to increase continuously until week 40 for both sexes. 

## 4. Discussion 

Various factors influence the growth model performance including sample size and data structure such as time intervals between records as well as confounding or environmental factors such as gender and pens or cages. The growth curves are different among species, among breeds within a species and vary among the individuals; therefore, they can be an interesting objective for genetic improvement. Currently, mink producers in Canada select their breeding animals based on the phenotypic performance for reproduction, growth traits (harvested weight and length) and fur quality, and therefore, understanding the biology of growth traits are important and useful for successful breeding programs. As expected, the mean values and their standard errors of body weight were increased with age except for week 23 that their weights were reduced compared to week 19 (Table 3). Sørensen et al. [13] reported lower values on the mature body weight of mink at 26 weeks of age (average of 2.76 kg and 1.36 kg for high feed efficiency males and females, respectively). This might be due to differences in the feed sources, management and color of the mink [12,15]. Notably, Sørensen et al. [13] used the standard brown mink while our study used the black mink. At the early stage of life (week 3–7), body weights were reported to be not significantly different between males and females mink [13,27]. Our results partially agreed with these results as we only observed significantly higher body weights in males compared to females at week 7 but not week 3 (Table S2). Approximately, 68% and 64% of the final body weights were gained from week 7 to week 15 for males and females, respectively (Table 3). These results were in agreement with the previous reports, which showed the majority of body weight gain was achieved from early July (week 7–8) to the end of September (week 15–16) [13,28]. However, it is important to note that chemical compositions and metabolic energy of mink diets were changed in weaning, growth and furring periods (Table 1). The highest metabolic energy per 100 grams of feed was given to mink during the period of 1 July 2018 (~week 7) to 13 August 2018 (~week 13) in order to adapt with high energy demands for growth at this period. Unfortunately, no information of nutrient was reported in the previous studies of growth models in mink. The Pearson correlations (0.89) between body weights at harvest (week 31) and body length at harvest in this study were slightly lower than the value (0.92) reported by Liu et al. [29] but higher than the values ( 0.75 in male and 0.73 in females) reported by Thirstrup et al. [30]. Interestingly, the correlation between body weight at week 11 and harvest body length was 0.86, which implies that there is a possibility to select mink with longer body length based on the body weight of animals at the beginning of the growth period. The correlation between body weight and size depends on the development stage of mink as Nielsen et al. [31] suggested that selection for August weight produces lean mink while selection for November weight produces fat mink [31]. Selection for higher body weight might not be the main target for the mink farmers since it encounters reproduction and pelt quality traits [30,32].

The correct choice of model is essential for understanding the animal growth as choosing a poor-fitting model can lead to unrealistic growth curves and consequently biologically meaningless growth rates, inflection points, upper asymptotes, and other parameter values. Previously, Liu et al. [12] reported that Gompertz, Logistic, and Richards were the best models for fitting the mink data according to AIC and BIC criteria. Similarly, we reported that Logistic and Richards were among the best models for male data; however, Gompertz model was not among the best models in the current study. Notably, Liu et al. [12] used a fewer number of models (six) compared to ten models in our study. Moreover, estimation of the goodness of fit of the models depends on the data used in the study. Liu et al. [12] used a fewer mink (300 mink) compared to our study and they fitted models separately for each of five color types (standard black, brown, mahogany, Hedlund white, and sapphire), and thus fewer mink were used for model performance test of each color compared to our study. However, Liu et al. [12] used a shorter interval for measuring body weight (once a week), that might result in more appropriate description of models. In our study, mink were weighed manually which required an extensive laboring and therefore longer measuring intervals of four weeks were adapted here. In this study, Richards model was the second best model for males and was the best model for females. Richards model uses sigmoid functions but adding more parameters, which allows modelling more flexible S-shaped curves [33,34,35]. It is widely used for modelling growth curve because of its flexibility and it can be transformed to other three-parameter models by fixing the parameter m in its equation. When m = −1, m = 1 or m = −1/3, Richards will be similar as Brody, Logistic or von Bertalanffy models, respectively. The estimated values of 1.03 for m parameter in Richards model in male data (Table 5) indicated that Richards model had close performance to Logistic model. The Richards model was also reported to be superior to other models for body mass in several studies in birds [35,36]. The same values of AIC and BIC were obtained for Weibull, Bridges and Janoscheck models in the current study. García-Muñiz et al. [37] also reported the same values of AIC and BIC for these growth curves models in goat. In fact, all Weibull, Bridges, and Janoscheck models use the Weibull distribution; however, Bridges and Janoscheck models have an initial body weight parameter for describing the postnatal growth of individuals. Similar performance reported for Weibull, Bridges and Janoscheck model in the current study might reflect that adding an initial body weight had a little effect on the growth curves of mink. To the best of our knowledge, no study has been devoted to test the polynomial models in mink. In minipig, Kohn et al. [10] showed that third and fourth degrees of linear polynomials had better performance than three and four parameter non-linear growth models. The non-linear models had worse performance than polynomial models in Kohn et al. [10] that might be due to that the authors did not observe far enough into the right asymptote. The differences in the model ranking in Kohn et al. [10] compared to ours might be mainly due to the differences in the growth patterns of mink and minipig. Additionally, Kohn et al. [10] compared the growth curve of minipig and fastening of commercial pigs and indicated that growth curve in minipig had less sigmoid than fastening pigs. 

The estimated growth parameters not only varied by the models but also varied among different production lines or different color types in mink. For instance, Sørensen et al. [13] showed that the estimates for maximum weight (*a*) in brown mink were 2.56 kg and 2.96 kg for high and low feed efficiency lines using Logistic function, respectively. Liu et al. [12] reported that the maximum weight ranged from 2.69 kg in sapphire mink to 3 kg in brown mink using the Logistic model. Sørensen et al. [13] also observed the differences in growth patterns of males and females. These differences might be due to higher fat than protein deposition in early August in females [31]. The growth curves obtained from Richards model (Figure 1) reflected the pattern obtained from the actual data as the major growth took place from week 7 to week 15 and the maximum growth was archived around week 18 for females and around week 20 for males. The major increase in the body weight during week 7–week 15 was mainly caused by deposition of protein, growth of skeleton, and development of body length and summer coat [13]. After week 18, the major change in metabolism is occurred since mink start to deposit more fat than protein [13] and develop the winter coat (pelting periods), and thus they increased their body weights slowly or remained the same body weights. Previous studies indicated that body weight and growth curve shape varied among individuals and between high and low feed efficient lines [13,38]. High feed efficient mink had shifted the growth curve to the right [13], therefore it might be possible to select the mink with higher feed efficient based on the shape of the growth curve. In addition to the fur quality and reproductive performance, feed efficiency is among the most interested traits for mink farmers [28]. Selection for feed efficient mink might need individual caging, which allows optimizing the nutrient for male and female mink that have different growth patterns. Although, mink nutrients can be adjusted for optimizing their growth performance, it is challenging because mink feed source depends on the availability of the by-products from human food production. Nevertheless, further heritability estimation of growth curve parameters and their genetic correlations with other economically important traits could be useful for future implementation of genetic/genomic selection for improvement of body shape (weight and/or length) based on growth curve parameters. Moreover, the genetic mapping in mink is necessary to better understand the biology underlying growth traits to increase the accuracy of genomic prediction if applied [39,40,41]. In this study, Richards model was suggested for modelling growth in mink but it might be required to be validated in other farms with more records before implementing in the future genetic or genomic selection programs. 

## 5. Conclusions 

This study showed the possibility of using four-parameter growth models for modelling growth in the Canadian mink populations. Large variation obtained from growth curve parameters suggested the necessity of understanding genetic parameters for growth curve parameters in order to develop the genetic or genomic selection programs based on the shape of the growth curves. 

## Figures and Tables

**Table 1:** Chemical compositions and metabolic energy of mink diets at weaning, growth and furring periods.

Diets	May 6– May 20	May 21– Jun 5	Jun 6– Jun 30	July 1– Aug 13	Aug 14– Sep 10	Sep 11– Oct 31	Nov 1– Dec 10
Chemical compositions							
Dry matter (%)	38.62	37.42	40.23	41.9	50.13	55.01	54.81
Fat (%)	20.1	23.82	26.68	29.3	23.39	20.84	24.68
Protein (%)	42.78	42.03	41.28	37.27	40.58	42.09	37.5
Ash (%)	10.22	9.73	8.90	7.44	7.58	7.68	7.26
Metabolic energy *							
Gross Energy (Kcal/100g)	413	434	453	470	432	419	437
%ME/DP	39.6	37	34.9	30.3	35.9	38.4	32.8
%ME/DF	41.6	46.9	50.4	53.4	46.3	42.6	48.3
%ME/DCHO	18.8	16.1	14.7	16.3	17.8	19	18.9
Total (%)	100	100	100	100	100	100	100

* ME—metabolic energy, DP—digestible protein, DF—digestible fat, DCHO—digestible carbohydrate.

**Table 2:** Growth models used in the study.

Names	Equation	Numbers of Growth Curve Parameters	References
Logistic	$\mathrm{BWt}=\frac{a}{\beta \times \left(1+e^{-kt}\right)}$	3	[17]
Gompertz	$\mathrm{BWt}=a\times e^{-\beta \times e^{-kt}}$	3	[18]
von Bertalanffy	$\mathrm{BWt}=a\times \left(1-\beta \times e^{-kt}\right)$ ^3^	3	[19,25]
Brody	$\mathrm{BWt}=a\times \left(1-\beta \times e^{-kt}\right)$	3	[20]
Richards	$\mathrm{BWt}=\frac{a}{{\left(1-\beta \times e^{-kt}\right)}^{\frac{1}{m}}}$	4	[22]
Weibull	$\mathrm{BWt}=a-\beta \times e^{-kt^{m}}$	4	[21]
Bridges	$\mathrm{BWt}={\mathrm{BW}}_{0}+a\times \left(1-e^{-kt^{m}}\right)$	4	[23]
Janoscheck	$\mathrm{BWt}=a-(a-{\mathrm{BW}}_{0})\times e^{-kt^{m}}$	4	[24]
Third degree polynomial	BWt = d_0_ + d_1_ × t + d_2_ × t^2^ + d_3_ × t^3^	-	-
Fourth degree polynomial	BWt = d_0_ + d_1_ × t + d_2_ × t^2^ + d_3_ × t^3^ + d_4_ × t^4^	-	-

BWt—body weight in kg at the time t; BW_0_—initial body weight in kg; α—mature body weight in kg; t—age in weeks; β, k and m—parameters specific for the function; β characterizes the first part of growth before the point of inflection; k describes the second part in which growth rate decreases until the animal reaches the asymptotic or mature weight (α), m is the shape parameter determining the position of the curve point inflection, d_0_—the model intercept, d_1_–d_4_—the regression coefficients.

**Table 3:** Descriptive statistics for body weights of American mink males and females from week 3 to week 31.

Week	Males			Females			Correlation with Harvest Body Length **
	N	BW * (±SE)	Range	N	BW (±SE)	Range	
3	359	0.15 ± 0.02	0.10–0.20	354	0.13 ± 0.01	0.10–0.18	0.51 ± 0.03
7	359	0.56 ± 0.10	0.28–0.93	354	0.47 ± 0.08	0.23–0.73	0.44 ± 0.03
11	358	1.72 ± 0.15	1.08–2.33	352	1.15 ± 0.12	0.86–1.81	0.86 ± 0.02
15	358	2.67 ± 0.26	1.37–3.33	351	1.53 ± 0.19	1.11–2.78	0.88 ± 0.02
19	359	2.92 ± 0.33	1.37–3.63	352	1.63 ± 0.20	1.06–2.65	0.88 ± 0.02
23	358	2.89 ± 0.36	1.25–3.98	348	1.61 ± 0.21	1.09–2.71	0.88 ± 0.02
27	352	3.01 ± 0.35	1.74–3.95	341	1.62 ± 0.19	1.02–2.23	0.86 ± 0.02
31	347	3.10 ± 0.36	1.57–4.10	335	1.62 ± 0.19	1.02–2.26	0.89 ± 0.02

* body weight measure based on kg; ** Pearson correlation and standard errors of correlation of body weight at the week of measure with the body length at harvest.

**Table 4:** The goodness of fit of growth curve models for body weights in American mink males and females.

Model	Males		Females	
	AIC *	BIC **	AIC	BIC
Logistic	756.21	780.03	−1576.61	−1552.86
Gompertz	872.47	896.29	−1417.92	−1394.18
von Bertalanffy	947.17	970.1	−1351.65	−1327.90
Brody	2358.16	2381.98	−561.43	−537.68
Richards	758.15	787.93	−1587.20	−1557.52
Weibull	782.68	812.45	−1587.19	−1557.51
Bridges	782.68	812.45	−1587.19	−1557.51
Janoscheck	782.68	812.45	−1587.19	−1557.51
Third degree polynomial	1952.43	1982.21	−931.44	−901.76
Fourth degree polynomial	990.04	1025.77	−1399.12	−1363.51

* AIC—Akaike’s information criterion and ** BIC—Bayesian information criterion.

**Table 5:** Estimated parameters and their 95% confidence interval for ten growth curve models in American mink males and females.

Model	Parameters *	Males		Females	
		Estimate (±SE)	95% CI	Estimate (±SE)	95% CI
Logistic	α	3.00 ± 0.01	2.99–3.02	1.64 ± 0	1.63–1.65
	β	10.33 ± 0.04	10.25–10.40	9.03 ± 0.05	8.93–9.12
	k	2.31 ± 0.04	2.24–2.39	2.28 ± 0.04	2.20–2.37
Gompertz	α	3.05 ± 0.01	3.03–3.06	1.65 ± 0.01	1.64–1.66
	β	13.19 ± 0.56	12.07–14.45	10.55 ± 0.56	9.34–12.01
	k	0.75 ± 0	0.74–0.75	0.73 ± 0	0.72–0.74
von Bertalanffy	α	3.07 ± 0.01	3.05–3.09	1.65 ± 0.01	1.64–1.66
	β	2.36 ± 0.7	2.21–2.51	2.61 ± 0.14	2.44–2.76
	k	0.24 ± 0	0.24–0.25	0.29 ± 0.01	0.28–0.30
Brody	α	3.49 ± 0.03	3.44–3.54	1.77 ± 0.01	1.75–1.79
	β	1.35 ± 0.01	1.32–1.37	1.41 ± 0.02	1.38–1.45
	k	0.09 ± 0	0.09–0.10	0.12 ± 0	0.12–0.13
Richards	α	3.00 ± 0.01	2.99–3.02	1.63 ± 0.01	1.62–1.64
	β	94.19 ± 31.66	47.82–192.14	247.62 ± 129.58	99.25–747.99
	k	0.44 ± 0.02	0.40–0.48	0.53 ± 0.03	0.47–0.60
	m	1.03 ± 0.11	0.82–1.26	1.58 ± 0.20	1.24–1.99
Weibull	α	2.98 ± 0.01	2.97–3.01	1.63 ± 0	1.62–1.64
	β	2.88 ± 0.02	2.84–2.92	1.53 ± 0.01	1.50–1.55
	k	−8.08 ± 0.16	−8.42–7.75	−7.32 ± 0.20	−7.72–6.94
	m	3.28 ± 0.07	3.15–3.42	3.11 ± 0.08	2.95–3.27
Bridges	BW_0_	0.10 ± 0.02	0.07–0.14	0.10 ± 0.01	0.08–0.12
	α	2.88 ± 0.02	2.84–2.92	1.53 ± 0.01	1.50–1.55
	k	7 × 10^−5^ ± 13 × 10^−5^	4 × 10^−5^–9 × 10^−5^	3 x 10^−5^ ± 5 × 10^−5^	2× 10^−5^–4 × 10^−5^
	m	3.28 ± 0.07	3.15–3.42	3.11 ± 0.08	2.95–3.27
Janoscheck	BW_0_	0.10 ± 0.02	0.07–0.14	0.10 ± 0.01	0.08–0.12
	α	2.98 ± 0.01	2.97–3.0	1.63 ± 0	1.62–1.64
	k	3 × 10^−5^ ± 5 × 10^−5^	2 × 10^−5^–4 × 10^−5^	7 × 10^−5^ ± 13 × 10^−5^	4 × 10^−5^–10 × 10^−5^
	m	3.28 ± 0.07	3.15–3.42	3.11 ± 0.08	2.95–3.27

* α—mature body weight in kg; BW_0_—initial body weight in kg; β, k, and m—parameters specific for the function. β characterizes the first part of growth, before the point of inflection, and k describes the second part, in which growth rate decreases until the animal reaches the asymptotic or mature weight (α), m is the shape parameter determining the position of the inflection of the curve point; CI—confidence interval.

## Supplementary Materials

The following are available online at https://www.mdpi.com/2076-2615/10/1/22/s1, Table S1: Feed ingredients in different growth periods of mink. Table S2. Estimated least square means of body weight by weeks using a liner mixed model. 2a. Estimated least square means for fixed effects on a linear mixed model, 2b. Differences in least square means for fixed effects on a linear mixed model.
